# Real-World Healthcare Resource Use Associated with Recurrent or Metastatic Head and Neck Cancer Patients Care in Portugal—TRACE Study

**DOI:** 10.3390/curroncol31080318

**Published:** 2024-07-26

**Authors:** Maria Margarida Teixeira, João Dias, Teresa André, Ana Joaquim, Ricardo Fernandes, Joana Magalhães, Laura Marreiros, Leonor Pinto, Leonor Ribeiro, Mafalda Nogueira, Catarina Morais

**Affiliations:** 1 Instituto Português de Oncologia de Coimbra Francisco Gentil, 3000-075 Coimbra, Portugal; 2 Instituto Português de Oncologia do Porto Francisco Gentil, 4200-072 Porto, Portugal; 3 Hospital Dr. Nélio Mendonça, 9000-177 Funchal, Portugal; 4 Unidade Local de Saúde de Gaia/Espinho, 4434-502 Vila Nova de Gaia, Portugal; 5 Unidade Local de Saúde de Braga, 4710-243 Braga, Portugal; 6 Unidade Local de Saúde do Algarve, 8000-386 Faro, Portugal; 7 Unidade Local de Saúde de Almada-Seixal, 2805-267 Almada, Portugal; 8 Unidade Local de Saúde de Coimbra, 3004-561 Coimbra, Portugal; 9 Unidade Local de Saúde de Santa Maria, 1649-035 Lisbon, Portugal; 10 MSD Portugal, 2770-192 Paço de Arcos, Portugal

**Keywords:** head and neck, recurrent or metastatic disease, real-world, healthcare resources, patient management

## Abstract

Recurrent or metastatic head and neck squamous cell carcinoma (R/M HNSCC) is a challenging disease, requiring personalized management by a multidisciplinary team. The aim of this retrospective multicentric study was to characterize real-world healthcare resource use and patient care for R/M HNSCC in Portugal during the first year after diagnosis. A total of 377 patients ineligible for curative treatment were included, mostly male (92.8%), aged 50–69 years (74.5%), with heavy alcohol (72.7%) or smoking habits (89.3%). Oropharynx (33.2%) and oral cavity (28.7%) were primary tumor locations, with lung metastases being the most common (61.4%). Eligible patients for systemic treatment with palliative intent (80.6%) received up to four treatment lines, with varied regimens. Platinum-based combination chemotherapy dominated first-line treatment (>70%), while single-agent chemotherapy and anti-PD1 immunotherapy were prevalent in later lines. Treatment approaches were uniform across disease stages and primary tumor locations but varied geographically. Treated patients received more multidisciplinary support than those who were ineligible. This study provides the first Portuguese real-world description of R/M HNSCC patient characteristics, treatment patterns, and supportive care during the year after diagnosis, highlighting population heterogeneity and aiming to improve patient management.

## 1. Introduction

Head and neck cancer is the fifth most common malignancy worldwide and the sixth most common cause of cancer-related death [1]. More than 90% of these tumors are squamous cell carcinomas (HNSCC) [1], a heterogeneous variety of malignant tumors originating from epithelial cells of the oral cavity, oropharynx, nasopharynx, hypopharynx, and larynx [2,3]. Each year, approximately 900,000 new HNSCC cases are diagnosed and over 400,000 deaths are recorded, with the incidence projected to increase by 30% by 2030 [4]. In Portugal, 2424 new HNSCC cases and 1103 annual deaths were reported in 2020, representing 4.0% of all new cases and 3.7% of deaths [4]. Several environmental and lifestyle risk factors are strongly associated with HNSCC, namely tobacco and alcohol consumption, behaviors that are responsible for over 75% of cases [5,6].

The stage at diagnosis guides the management of HNSCC patients and predicts survival rates. Most HNSCCs are at an advanced and metastatic stage (III or IV) at diagnosis, involving regional nodes and/or distant metastases [6,7]. Half of the patients submitted to primary treatment with curative intent will eventually recur with distant metastases and/or locoregional disease and die [8]. Thus, the prognosis for recurrent or metastatic (R/M) HNSCC is notably poor, and the disease is, therefore, incurable. Life expectancy can reach 15 months when the best first-line therapy available is feasible [9].

Until recently, treatment options for R/M HNSCC were limited and consisted of chemotherapy and/or cetuximab, namely a combination of platinum-based agents (cisplatin or carboplatin) and 5-fluorouracil (5-FU) with cetuximab (anti-EGFR monoclonal antibody). With the emergence of immunotherapy, novel targeted agents for HNSCC are now available with the most awaited results and improved patient’s overall survival [9,10,11,12,13,14]. The programmed cell death (PD-1) immune checkpoint inhibitor pembrolizumab became the first-line standard of care for patients with R/M disease, PD-L1 positive, who have no surgical or radiotherapeutic option, either as monotherapy or in combination with platinum-based agents (cisplatin or carboplatin) plus 5-FU [7,12,15]. Nevertheless, it is only recently that guidelines have clearly defined the standard of care for first-line treatment of R/M HNSCC and a therapeutic pathway for this complex disease [7,15].

R/M HNSCC patient care is also a challenging and sensitive subject. These patients have specific and complex needs, due to both the disease and the effects of treatment, as they may experience pain, cough, speech and swallowing dysfunction, weight loss, and dental problems [7,16]. A multidisciplinary team approach is needed to address clinical care, rehabilitation, and social needs: it evaluates the impact of the disease and its treatments on patients’ lives and makes informed decisions on clinical care and rehabilitation services for patients [7].

Notwithstanding the available publications on the real-world treatment and care of R/M HNSCC patients worldwide [14,17,18,19,20,21], the Portuguese reality has never been documented. In order to fill this gap, the TRACE study primarily aimed to characterize real-world healthcare resource use (HCRU) in Portugal related to R/M HNSCC patient care during the first year after diagnosis of advanced disease, in patients who were ineligible for curative treatment. Additionally, it intended to describe the demographic and clinical characteristics of Portuguese R/M HNSCC patients, as well as their treatment patterns. The results of this real-world study may contribute to further explaining the therapeutic value of the different treatment modalities in the clinical practice setting and help policymakers and healthcare providers to make informed decisions on how to improve the management of R/M HNSCC patients in Portugal.

## 2. Materials and Methods

### 2.1. Study Design

TRACE was a multicentric, retrospective, cross-sectional study of R/M HNSCC patients, conducted in nine Portuguese hospitals (*Instituto Português de Oncologia do Porto Francisco Gentil, Instituto Português de Oncologia de Coimbra Francisco Gentil, Unidade Local de Saúde de Gaia/Espinho, Unidade Local de Saúde de Braga, Unidade Local de Saúde de Coimbra, Unidade Local de Saúde de Santa Maria, Unidade Local de Saúde de Almada-Seixal, Unidade Local de Saúde do Algarve*, and *Hospital Dr. Nélio Mendonça*) from different geographical locations and of different typologies (2 cancer centers, 4 university general hospitals, and 3 non-university hospitals). It was conducted between July 2020 and June 2022.

### 2.2. Study Participants

R/M HNSCC patients were included in the study if all the following inclusion criteria were met: adult patients (aged ≥18 years) diagnosed with R/M HNSCC ineligible for curative treatment between 1 June 2017 and 31 December 2019, with primary tumors in the oral cavity, oropharynx, hypopharynx, larynx, or other, and ill-defined sites in the lip, oral cavity, and pharynx.

Patients younger than 18 years of age, with an unconfirmed R/M HNSCC diagnosis or confirmed only at autopsy, or with carcinoma in other locations, such as the nasopharynx, nasal cavity, and paranasal sinuses or salivary glands, were excluded from the study.

### 2.3. Study Objectives

The main objective of TRACE was to characterize real-world HCRU related to R/M HNSCC patient care in Portugal. In addition, it aimed to describe patient’s demographics, risk factors, disease stage, primary tumor and metastasis locations, and treatment patterns.

### 2.4. Data Sources/Measurement

This study was based on secondary data collection from electronic medical records that occurred during a 3-month period per participating center after the follow-up period of the last patient was complete. Data were collected for up to one year, since the diagnosis of R/M HNSCC, and until the end of the patient follow-up, death, or cut-off date. It contemplated the collection of sociodemographic (gender, age, and region), risk factors (smoking and heavy alcohol habits—defined as the ingestion of over 100 g of alcohol per day), clinical (disease stage, primary tumor and metastasis locations, and the Eastern Cooperative Oncology Group [ECOG] performance status score), and HCRU (treatment for R/M HNSCC, complementary exams, hospitalizations, consultations, and supportive care) data.

### 2.5. Statistical Analysis

Quantitative variables were characterized using descriptive statistics as mean and standard deviation (SD) values. Qualitative variables were summarized using absolute and relative frequencies.

Exploratory inference analysis was conducted for chemotherapy, cetuximab, immunotherapy, radiotherapy, and surgery according to geographic region, disease stage, and primary tumor location subgroups.

Parametric statistical methods were used whenever possible. If data had a non-Gaussian distribution, non-parametric statistical methods were used. Comparison of continuous variables between two or more groups was performed by ANOVA or Kruskal–Walli’s test, as appropriate. Comparison of categorical variables between groups was performed using the Fisher’s exact test.

All hypotheses were tested using two-sided tests at the ɑ = 0.05 significance level. *p*-values were corrected for multiple comparisons with the Benjamini–Hochberg method, considering a false-discovery ratio of 0.05.

All computations were implemented in Python 3.6 but using statistical methods from R software (R 4.1.2) [22,23].

## 3. Results

### 3.1. Patient’s Sociodemographic and Clinical Characteristics

**Figure 1:**
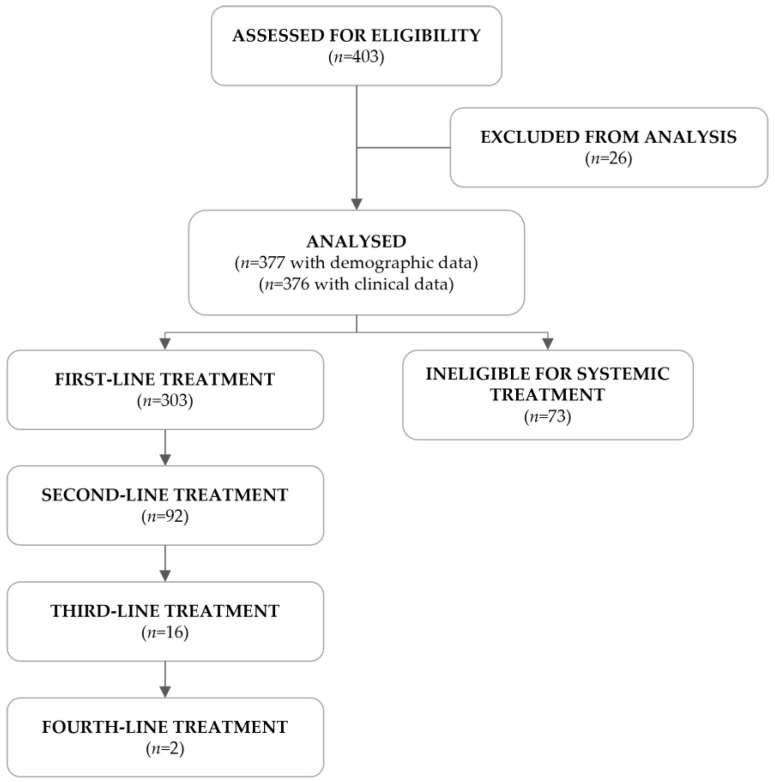
Flowchart of the study population.

A total of 403 R/M HNSCC patients were assessed for eligibility, of which 26 failed screening. The remaining 377 met the eligibility criteria and were included in this study (Figure 1). Of the 376 patients with clinical information, 303 (80.6%) were eligible for systemic treatment, as per clinical decision, and received at least one line of treatment, whereas the remaining 73 (19.4%) were ineligible.

The sociodemographic and clinical characteristics of the study population are presented in Table 1 and Table S1.

R/M HNSCC patients were predominantly male (92.8%), aged between 50 and 69 years (74.5%), and geographically evenly distributed across the north (39.8%), center (30.8%), and south/islands (29.4%) of Portugal. Tobacco and alcohol use were common in this cohort, with 89.3% of the patients being current or former smokers and 72.7% having heavy alcohol consumption habits. Moreover, patients ineligible for systemic treatment (67.1%) had a higher tendency for current tobacco use than those receiving systemic treatment (53.2%), although not statistically significant (*p* = 0.1014), while alcohol consumption was similar between groups (*p* = 0.7064).

At diagnosis, 234 patients (62.2%) were documented with metastatic disease and 142 (37.8%) with recurrent, of which 38 (26.8%) also had identified metastasis. Primary tumors were mainly located in the oropharynx (33.2%) or in the oral cavity (28.7%), and the lung was the most common site of metastasis (61.4%). Of note, 90 patients (34.1%) had multiple metastatic sites. The performance status of R/M HNSCC patients at diagnosis was assessed using the ECOG performance status score and ranged from an ECOG score of 0 to 3, with the majority of individuals having a score of 1 (60.7%). In fact, patients ineligible for systemic treatment displayed significantly higher ECOG performance status scores (≥2: 72.2%) than those eligible (≥2: 17.2%; *p* < 0.0001) (Table S1).

### 3.2. Overall Use of Healthcare Resources

In line with the primary objective of this study, the total use of healthcare resources associated with the care of R/M HNSCC patients during the one-year follow-up after diagnosis was evaluated (Table 2). On average, patients were followed for 8.2 ± 4.0 months, of which 164 (43.6%) reached the one-year mark.

Most patients received chemotherapy (73.7%) and cetuximab (43.9%) as systemic treatment, whereas only a proportion were prescribed anti-PD1 immunotherapy (13.8%). In the study population, 95.5% received concomitant medication, 26.3% underwent radiotherapy, and 9.3% underwent surgery. Nearly all patients performed laboratory tests (96.5%) and imaging assessments (92.0%), and 47.1% underwent other complementary exams. Also, 68.1% of the individuals were hospitalized at least once, and 98.7% attended at least one consultation after treatment initiation, either with a healthcare specialist (outpatient: 94.4%), an emergency consultation (59.3%), or of supportive care (nutritional support: 51.6%; psychological treatment: 19.4%; speech therapy: 6.4%).

### 3.3. Treatment Patterns

Of the 376 patients in this study, 303 received at least one line of treatment, and 73 were considered ineligible for chemotherapy, cetuximab, or anti-PD1 immunotherapy but underwent radiotherapy (24.7%) or surgery (4.1%).

Among those who received at least one line of treatment, a decreasing number of patients proceeded to a subsequent line, with only two reaching the fourth-line setting. After completing treatment, 286 patients continued to be followed at the participating center, of whom 8.4% underwent radiotherapy and 1.8% underwent surgery. The remaining 17 patients were lost to follow-up, died, or reached the cut-off date.

**Figure 2:**
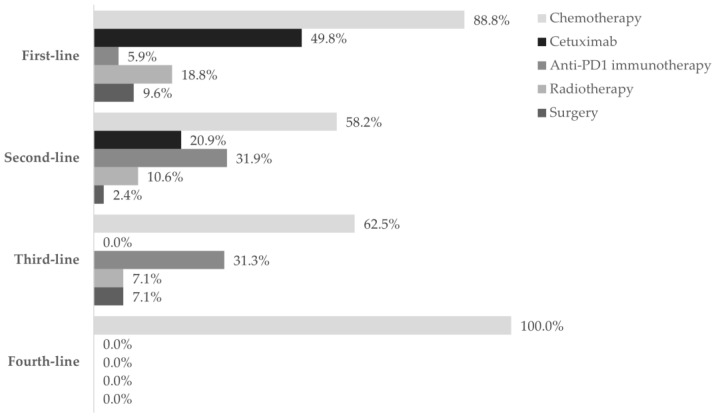
The utilization of treatment modalities across four lines of treatment. Frequency of patients using chemotherapy (■), cetuximab (■), anti-PD1 immunotherapy (■), radiotherapy (■), and surgery (■) in each of the four lines settings, during the one-year follow-up after diagnosis of R/M disease. Data are shown as the relative frequency of patients receiving each modality, calculated within the total in each treatment line (first line: *n* = 303; second line: *n* = 91; third line: *n* = 16; fourth line: *n* = 2).

**Figure 3:**
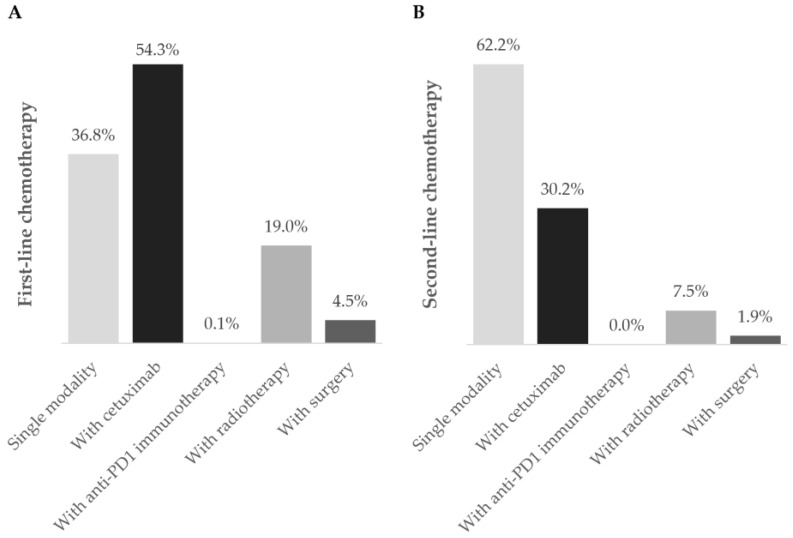
Chemotherapy regimens during the (**A**) first- and (**B**) second-line settings. Frequency of patients using chemotherapy as a single modality (■) or in combination with cetuximab (■), anti-PD1 immunotherapy (■), radiotherapy (■), and surgery (■) during first- and second-line treatment during the one-year follow-up after diagnosis of R/M disease. Data are shown as the relative frequency of patients, calculated within the total receiving chemotherapy in each treatment line (first line: *n* = 269; second line: *n* = 53).

**Figure 4:**
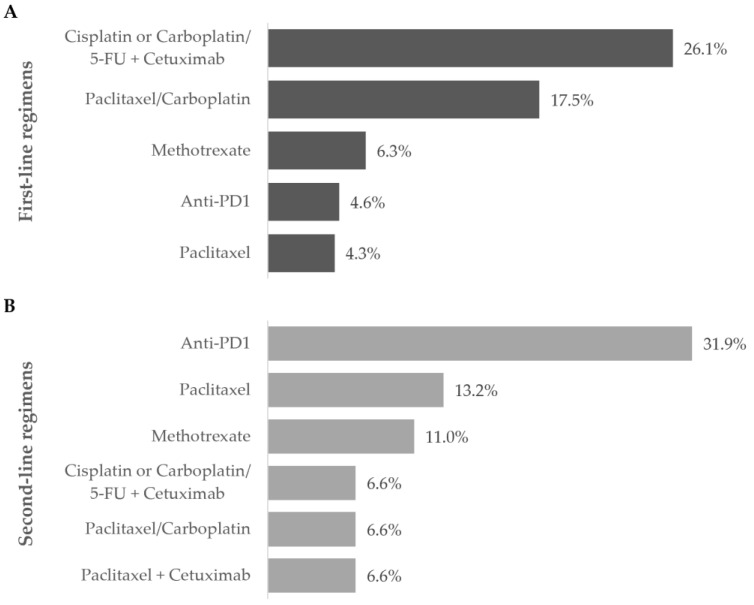
Most frequent treatment regimens during the (**A**) first- and (**B**) second-line settings. Frequency of patients receiving the most commonly prescribed regimens during the first and second line of treatment during the one-year follow-up after diagnosis of R/M disease. Data are shown as the relative frequency of patients, calculated within the total in each treatment line (first line: *n* = 303; second line: *n* = 91).

The first-line treatment started at a mean of 6.6 ± 16.6 weeks after treatment decision and lasted for 17.4 ± 13.5 weeks, on average. The second, third, and fourth lines lasted 11.3 ± 11.8, 8.0 ± 4.2, and 6.0 ± 2.0 weeks, on average, respectively. Multiple treatment regimens were applied across different therapeutic lines: 40 in the first-line, 16 in the second-line, 7 in the third-line, and 2 in the fourth-line setting. Figure 2, Figure 3 and Figure 4 and Table S2 detail information on the different modalities per line of treatment; Table S3 shows the duration, number of cycles/procedures, and dose of each modality per line of treatment.

#### 3.3.1. First Line

Chemotherapy was the prevailing first-line treatment modality (*n* = 269, 88.8%). It was mainly administered using carboplatin (129, 48.0%), 5-FU (124, 46.1%), paclitaxel (97, 36.0%), and cisplatin (95, 35.3%) as a single- (69, 25.6%), two- (183, 68.0%), or three-agent (17, 6.3%) regimen. Chemotherapy was mostly combined with cetuximab (146, 54.3%) and, to a lesser extent, with radiotherapy (51, 19.0%), surgery (12, 4.5%), and/or immunotherapy (2, 0.1%). Cetuximab was also frequently used (151, 49.8%) and associated with chemotherapy in 146 (96.7%) patients.

As for anti-PD1 immunotherapy, 18 patients (5.9%) received this treatment, of which 14 (77.8%) were treated with it as a single regimen.

Fifty-seven patients (18.8%) underwent radiotherapy and 29 (9.6%) underwent surgery. Both modalities were generally associated with chemotherapy (radiotherapy: 51, 89.5%; surgery: 17, 58.6%).

Overall, the most frequent first-line treatment modality was the combination of cisplatin or carboplatin plus 5-FU with cetuximab (79, 26.1%).

The use of each of these healthcare resources was evaluated by patient’s geographical region, disease stage, and primary tumor location (Figure S1). Chemotherapy and cetuximab were the only modalities revealing statistically significant differences, but only when evaluated by region. While chemotherapy was more frequently used in the north (92.2%) and center (95.7%) of Portugal than in the south/islands (78.1%; *p* = 0.002), cetuximab administration was higher in the north (58.3%) and in the south/islands (56.2%) compared to the center (32.6%; *p* = 0.002) (Figure S1A,B). Similarly, the use of different chemotherapeutic agents was different between regions. In the south/islands, there was a higher use of cisplatin (50.7%; north: 37.7%; center: 19.3%; *p* = 0.001) and 5-FU (61.3%; north: 47.2%; center: 31.8%; *p* = 0.003). On the contrary, more patients from the center were prescribed paclitaxel (47.7%; north: 41.5%; south/islands: 17.3%; *p* = 0.001) and methotrexate (20.4%; north: 0.9%; south/islands: 0.0%; *p* = 0.001). The use of carboplatin (*p* = 0.088), docetaxel (*p* = 0.531), and gemcitabine (*p* = 0.185) was statistically similar across geographical regions.

#### 3.3.2. Second Line

Ninety-two patients required second-line treatment, of whom one had missing treatment data. Chemotherapy continued as the prevailing treatment modality (53, 58.2%), which was administered alone in 33 patients (62.2%). The most frequent chemotherapeutic agent was paclitaxel (29, 54.7%), and patients were mostly prescribed single-agent therapy (36, 67.9%). More than half of patients (36, 67.9%) who received second-line chemotherapy switched from their previous chemotherapy regimen. Of the 19 (20.9%) patients who received cetuximab as targeted therapy, 16 (84.2%) combined it with chemotherapy.

Anti-PD1 immunotherapy was prescribed to 29 patients (31.9%) as a single modality in all cases, representing the most frequent regimen in this line. Most patients have switched from previous-line chemotherapy with cisplatin (10, 34.5%) or carboplatin (4, 13.8%) with 5-FU plus cetuximab, or paclitaxel with carboplatin (4, 13.8%).

Also, nine patients (10.6%) underwent radiotherapy and two (2.4%) underwent surgery.

#### 3.3.3. Third and Fourth Line

Of the 16 patients who received third-line treatment, 10 (62.5%) were treated with chemotherapy alone, 5 (31.3%) with anti-PD1 immunotherapy, 1 (7.1%) with radiotherapy, and 1 (7.1%) with surgery. Further, 6 of the 10 (60.0%) patients who received chemotherapy switched from previous anti-PD1 immunotherapy; 4 of the 5 (80.0%) patients who received anti-PD1 immunotherapy switched from previous chemotherapy.

The two patients who reached fourth-line treatment were treated only with chemotherapy: one with paclitaxel and the other with methotrexate.

### 3.4. Complementary Exams, Consultations, and Supportive Care

During the one-year follow-up period, patients underwent complementary exams, were hospitalized, attended consultations with healthcare specialists, or received some form of supportive care. The total use of each of these healthcare resources is specified above (Table 2); the use of these resources per line of treatment and by patients ineligible for treatment is shown in Table 3.

Overall, laboratory and imaging assessments were performed in the majority of patients. The use of the latter decreased across treatment lines and after discontinuation. Of note, PD-L1 status was assessed in 34 patients (9.0%) during systemic treatment and in 2 (0.5%) who were ineligible for treatment. However, these test results were not collected under the scope of this study.

The vast majority of this study population also attended outpatient consultations, namely with oncology specialists, and were prescribed concomitant medication. The use of multidisciplinary consultations was comparable between patients in the first-line setting (62.0%) and those ineligible for systemic treatment (66.7%) but decreased across treatment lines and after treatment discontinuation.

A higher frequency of patients ineligible for systemic treatment registered more hospitalizations (76.7%) and emergency consultations (57.5%) than those under treatment (hospitalizations: 44.9%; emergency consultations: 49.5%). The former also had longer hospital stays (mean days/patient = 43.7 ± 85.3) than patients during first-line (mean days/patient = 19.1 ± 21.8), second-line (mean days/patient = 14.9 ± 14.1), third-line (mean days/patient = 12.5 ± 3.5), and after systemic treatment discontinuation (mean days/patient = 18.4 ± 20.4). Conversely, access to nutritional support, psychological treatment, and speech therapy was higher among those receiving systemic treatment. Other supportive care measures, such as pain therapy, palliative care, stomal therapy, breathing rehabilitation, or social support, were also adopted, mainly during the first-line setting (26.7%) and in treatment-ineligible patients (38.4%).

## 4. Discussion

This retrospective, cross-sectional, and multicentric study is the first conducted in Portugal that characterizes real-world HCRU in the care of R/M HNSCC patients, during the first year following diagnosis of recurrent or metastatic disease. The demographic and clinical characteristics of Portuguese patients with advanced disease and the patterns of treatment were also described in this study.

Globally, this study sample was predominantly male (92.8%), with heavy alcohol consumption habits (72.7%), current or former smokers (89.3%), conserved ability to perform daily activities (ECOG performance status score ≤ 1: 72.2%), presenting with metastatic disease at diagnosis (62.2%), and primary tumors more frequently located in the oropharynx (33.2%). These cohort characteristics are similar to those documented in other European and North American studies [17,18,24]. While recent data emphasize human papillomavirus (HPV) as a strong prognostic factor in oropharyngeal HNSCC [25,26], HPV status was not commonly evaluated at the time of patient’s follow-up (July 2017–December 2020)—around 35% of patients with oropharynx-located HNSCC were tested for p16 and not included in the analysis. Nevertheless, despite guidelines recommending a similar treatment strategy for HPV-positive and -negative HNSCC tumors, evaluating HPV status is essential for the correct diagnosis and staging of oropharyngeal tumors [15].

Following the diagnosis of R/M disease, HNSCC patients were divided into two groups: eligible (80.6%) or ineligible (19.4%) for systemic treatment with palliative intent. Those eligible received up to four lines of treatment, with a significant number of different regimens registered across lines, consistent with previous R/M HNSCC studies conducted in Europe and the United States [17,18,19]. Ideally, this may reflect individualized treatment options that are based on the primary tumor location, disease stage, patient’s performance status, and therapeutic goals [15,17].

In this study, platinum-based combination chemotherapy regimens were most widely used as first-line treatment (over 70% of patients), while single-agent chemotherapy was more frequent in the second-line setting (39% of patients). R/M HNSCC patients from the United Kingdom and the United States received similar treatment between 2011 and 2014 [17,18]: around 75% of patients received platinum-based regimens during first-line treatment and single-agent chemotherapy regimens during second-line treatment. Concerning anti-PD1 immunotherapy, in the second (31.9%) and third line (31.3%), this modality was more frequent compared to the first-line setting, regardless of disease stage or tumor location. This was consistent with the standard of care and regulatory approval/reimbursement available at the time of patients’ follow-up, as the majority of patients were prescribed first-line platinum-based combination therapy and either single-agent chemotherapy regimens or anti-PD1 immunotherapy in the subsequent lines [27,28]. Presently, guidelines establish anti-PD1 immunotherapy as the standard of care in the first-line setting for R/M HNSCC patients [7,12,15]. In particular, patients with PD-L1-positive tumors benefit from anti-PD1 pembrolizumab immunotherapy (used as monotherapy or combined with cisplatin or carboplatin plus 5-FU) and have better overall survival than those under chemotherapy plus cetuximab [12,15]. Unfortunately, PD-L1 testing was not the standard of care when patients from this study were diagnosed with R/M HNSCC disease, and only a small percentage (nearly 10%, *n* = 34) assessed tumor PD-L1 expression. Currently, all patients with R/M HNSCC who are eligible for systemic treatment in Portugal undergo PD-L1 testing, as per recent recommendations [7,15].

The results also suggest different approaches to the treatment of R/M HNSCC patients by geographical region. Chemotherapy was more prescribed in the center (95.7%) and north (92.2%) of Portugal, and cetuximab had a higher prevalence in the north (58.3%) and south/islands (56.2%). While more studies are needed to understand the variation in the use of systemic treatment modalities between regions, it is important to note that there is a significant disease heterogeneity in Portugal, which varies between regions, particularly when comparing urban and rural areas; patients from rural areas tend to be diagnosed with HNSCC at more advanced stages. This heterogeneity directly affects the diversity of patients who are treated at each participating center, ultimately influencing the types of treatment approaches implemented.

Due to the advanced stage of the disease, patients with R/M HSNCC usually undergo surgery and radiotherapy for palliative purposes only. In this cohort, surgery (9.3%) was the least performed technique, likely due to the advanced and unresectable stage of tumors, and radiotherapy was mainly performed in patients ineligible for systemic treatment (24.7%).

The management of HNSCC patients requires a multidisciplinary team that is dedicated to the well-being of these patients at all stages of the treatment journey. Their goal is to evaluate the impact of the disease and of treatments on many aspects of daily living that negatively impact patient’s quality of life [16]. In this study, both eligible and ineligible patients for systemic treatment were submitted to complementary exams, hospitalized, attended consultations with healthcare specialists, or received some form of supportive care. Whereas nearly all patients took medication for disease management, there was considerable variation in the use of the other healthcare resources between groups. Ineligible patients had more hospitalizations (76.7%) and for longer periods, possibly due to a more debilitating condition (higher ECOG performance status scores). Patients receiving a first-line treatment attended a higher frequency of outpatient consultations (94.1%) and had more supportive care appointments, although the use of the latter decreased across several lines of treatment and after discontinuation. Similarly, La et al. reported less use of healthcare resources and supportive care measures after discontinuation of systemic treatment in metastatic HNSCC patients, namely of nutritional support (from 58.6%, after diagnosis of metastatic disease, to 49.4%, after discontinuation of treatment) and speech and swallowing therapy (from 22.3% to 9.2%) [17]. The authors suggested a shift in treatment strategies in order to maximize the patients’ time at home and with their families, which may also be the case here. Nevertheless, considering the patient’s well-being and the physical and psychological aggressiveness of the disease and its treatments, one cannot exclude the possibility that patients were receiving supportive care elsewhere or palliative care, or that this information may have been underreported by the investigators.

The limitations of this study must be considered. Due to the retrospective nature of this study, incomplete or missing data may have existed in medical records and must be acknowledged. For instance, some patients were prescribed medications as supportive care that were not documented in their medical records. Also, patients included in this study represent approximately 40% of all R/M HNSCC cases occurring in Portugal, which may have introduced a selection bias. A key limitation of this study arises from potential discrepancies among investigators in the interpretation of the electronic case report form regarding treatment lines. The divergent assumptions introduce potential bias in reported data, possibly inflating the documented number of treatment modalities. Unfortunately, the absence of specific treatment dates hinders the definitive confirmation of whether these interventions qualify as separate treatment lines or not.

## 5. Conclusions

This study provides original and important real-world data on the HCRU and treatment patterns of R/M HNSCC patients during the first year after diagnosis of recurrent or metastatic disease, in Portugal. The results show a high number of treatment modalities and the variation in the use of chemotherapy and cetuximab between geographical regions, corroborating the heterogeneity of the Portuguese R/M HNSCC patients. In addition, few patients received supportive care, which, if unintentional and not a result of underreporting, should be exploited to improve and provide adequate care to R/M HNSCC patients. The addition of this new layer of knowledge to the field may potentially guide future clinical practice guidelines that improve patient management. Future research should explore survival outcomes and health-related quality of life after systemic treatment, in the light of current guidelines, to better understand whether and where improvements need to be considered in the Portuguese clinical setting, namely in terms of resources, be they human, consumable, or financial.

## Figures and Tables

**Table 1:** Patients’ sociodemographic and clinical characteristics.

Sociodemographic Characteristics	*n* = 377
Gender, *n* (%)	
Male	350 (92.8)
Female	27 (7.2)
Age at study inclusion (years), *n* (%)	
18–49	30 (8.0)
50–59	156 (41.4)
60–69	125 (33.2)
≥70	66 (17.5)
Region, *n* (%)	
North	150 (39.8)
Center	116 (30.8)
South/Islands	111 (29.4)
Smoking status, *n* (%) (*Missing*: *n* = *2*)	
Current	209 (55.7)
Former	126 (33.6)
Never	40 (10.7)
Heavy alcohol consumption, *n* (%) (*Missing*: *n* = *3*)	
Yes	272 (72.7)
No	102 (27.3)
Clinical Characteristics	***n*** = **376**
Primary tumor location, *n* (%)	
Oropharynx	125 (33.2)
Lip/oral cavity	108 (28.7)
Hypopharynx	72 (19.1)
Larynx	65 (17.3)
Other	6 (1.6)
Disease stage at R/M diagnosis, *n* (%) (*Missing*: *n* = *1*)	
Metastatic *	234 (62.2)
Recurrent	142 (37.8)
Recurrent and metastatic	38 (26.8)
Metastasis location ^†^, *n* (%)	
Lung	162 (61.4)
Lymph nodes	91 (34.5)
Bone	46 (17.4)
Liver	22 (8.3)
Other	73 (27.7)
Nr of metastasis/patient ^†^, *n* (%)	
1	174 (65.9)
2	57 (21.6)
3	25 (9.5)
4	5 (1.9)
5	2 (0.8)
Widely metastasized	1 (0.4)
ECOG performance status score, *n* (%) (*Missing*: *n* = *2*)	
0	43 (11.5)
1	227 (60.7)
2	67 (17.9)
3	37 (9.9)

ECOG: Eastern Cooperative Oncology Group; R/M: recurrent/metastatic. * 8 metastatic patients without identified metastasis; ^†^ relative frequencies calculated in the 264 patients with identified metastatic location.

**Table 2:** Healthcare resource use in the care of R/M HNSCC patients over the one-year follow-up after diagnosis of R/M disease.

Type of HCR	*n* = 376
Medication, *n* (%)	
Concomitant medication	359 (95.5)
Chemotherapy	277 (73.7)
Cetuximab	165 (43.9)
Anti-PD1 immunotherapy	52 (13.8)
Non-medication, *n* (%)	
Radiotherapy	99 (26.3)
Surgery *	35 (9.3)
Exams	
Laboratory tests	363 (96.5)
Imaging assessments	345 (92.0)
ECG, biopsies, and other exams	176 (47.1)
Hospitalizations	256 (68.1)
Consultations	371 (98.7)
Outpatient consultation	355 (94.4)
Emergency consultation	223 (59.3)
Nutritional support	194 (51.6)
Psychological treatment	73 (19.4)
Speech therapy	24 (6.4)

ECG: electrocardiogram; HCR: healthcare resource. * Excision surgery, lymph node dissection, reconstructive surgery, and/or laser surgery.

**Table 3:** Exams, hospitalizations, consultations, and supportive care use by R/M HNSCC patients, who were eligible and ineligible for systemic treatment, during the one-year follow-up after diagnosis of R/M disease.

Type of HCR	First-Line (*n* = 303)	Second-Line (*n* = 92) ^‡^	Third-Line (*n* = 16) ^†^	Fourth-Line (*n* = 2)	After Discontinuation (*n* = 286)	Ineligible (*n* = 73)
Exams, *n* (%)						
Laboratory	295 (97.4) *	82 (96.5)	14 (100.0)	2 (100.0)	107 (37.5) *	62 (84.9)
Imaging	285 (94.4)	61 (71.8)	7 (50.0)	1 (50.0)	67 (23.5) *	51 (69.9)
ECG, biopsy, and others	134 (44.5) ^†^	13 (14.1)	3 (21.4)	0 (0.0)	25 (8.8) *	24 (32.9)
Outpatient consultations, *n* (%)	285 (94.1)	77 (90.6)	14 (100.0)	1 (50.0)	130 (45.8) ^†^	63 (86.3)
Oncology	274 (90.4)	75 (88.2)	14 (100.0)	1 (50.0)	100 (35.2) ^†^	47 (74.6)
Other specialties	213 (70.3)	42 (49.4)	7 (50.0)	1 (50.0)	84 (29.6) ^†^	43 (68.2)
Multidisciplinary	188 (62.0)	34 (40.0)	5 (35.7)	0 (0.0)	56 (19.7) ^†^	42 (66.7)
Emergency consultations, *n* (%)	150 (49.5)	30 (35.3)	4 (28.6)	0 (0.0)	59 (20.7) *	42 (57.5)
Hospitalizations, *n* (%)	136 (44.9)	21 (24.7)	3 (21.4)	0 (0.0)	101 (35.6) ^†^	56 (76.7)
Supportive care, *n* (%)						
Concomitant medication	282 (93.1)	68 (80.0)	11 (73.3)	2 (100.0)	143 (50.0)	68 (93.2)
Nutritional support	159 (52.5)	24 (28.2)	5 (35.7)	1 (50.0)	46 (16.1) *	21 (28.8)
Psychological treatment	46 (15.2)	10 (11.8)	1 (7.1)	0 (0.0)	22 (7.7) *	8 (11.0)
Speech therapy	16 (5.3)	7 (8.2)	2 (14.3)	0 (0.0)	3 (1.0) *	1 (1.4)
Other	81 (26.7)	15 (17.6)	2 (14.3)	0 (0.0)	56 (19.8) ^§^	28 (38.4)

ECG: electrocardiogram. * Missing = 1; ^†^ Missing = 2; ^‡^ Missing = 7; ^§^ Missing = 3.

## Data Availability

The raw data supporting the conclusions of this article will be made available by the authors, without undue reservation.

## Supplementary Materials

The following supporting information can be downloaded at: https://www.mdpi.com/article/10.3390/curroncol31080318/s1, Figure S1: Use of first-line chemotherapy, cetuximab, anti-PD1 immunotherapy, radiotherapy, and surgery by patient’s geographical region, disease stage, and primary tumor location; Table S1: Sociodemographic and clinical characteristics of patients eligible and ineligible for systemic treatment; Table S2: Chemotherapy regimens used as systemic treatment in R/M HNSCC patients during the one-year follow-up after diagnosis; Table S3: Duration, number of cycles/procedures, and dose of chemotherapy, cetuximab, anti-PD1 immunotherapy, radiotherapy, and surgery used in R/M HNSCC patients during the one-year follow-up after diagnosis.
