# Supplementary material for: Real-World Healthcare Resource Use Associated with Recurrent or Metastatic Head and Neck Cancer Patients Care in Portugal—TRACE Study
Source: Curr Oncol. 2024 Jul 26;31(8):4270–83. doi: 10.3390/curroncol31080318 (PMC11352379; doi:10.3390/curroncol31080318)
Supplement: Supplementary file 1 [file curroncol-31-00318-s001.zip › curroncol-3084033-supplementary.pdf]

Table S1. Sociodemographic and clinical characteristics of patients eligible and ineligible for systemic treatment.

Characteristics	Eligible (*n* = 303)	Ineligible (*n* = 73)	Unadjusted p-value [1]	Adjusted p-value [2]
**Gender, *n* (%)**			0.8963	0.8963
Male	282 (93.1)	67 (91.8)		
Female	21 (6.9)	6 (8.2)		
**Age at study inclusion (years), *n* (%)**			0.6708	0.7546
18-49	25 (8.3)	5 (6.8)		
50-59	128 (42.2)	27 (37.0)		
60-69	100 (33.0)	25 (34.2)		
≥70	50 (16.5)	16 (21.9)		
**Region, *n* (%)**			0.1375	0.2475
North	115 (38.0)	35 (47.9)		
Centre	92 (30.4)	23 (31.5)		
South/Islands	96 (31.7)	15 (20.5)		
**Smoking status, *n* (%)**	*Missing: n = 2*		0.0338	0.1014
Current	160 (53.2)	49 (67.1)		
Former	110 (36.5)	15 (20.5)		
Never	31 (10.3)	9 (12.3)		
**Heavy alcohol consumption, *n* (%)**	*Missing: n = 3*		0.4709	0.7064
Yes	215 (71.7)	56 (76.7)		
No	85 (28.3)	17 (23.3)		
**Primary tumor location, *n* (%)**			0.0595	0.1339
Oropharynx	100 (33.0)	25 (34.2)		
Lip/oral cavity	86 (28.4)	22 (30.1)		
Hypopharynx	52 (17.2)	20 (27.4)		
Larynx	59 (19.5)	6 (8.2)		
Other	6 (2.0)	0 (0.0)		
**Disease stage at R/M diagnosis, *n* (%)**			0.5779	0.7430
Metastatic	186 (61.4)	48 (65.8)		
Recurrent	117 (38.6)	25 (34.2)		
**Metastasis location, *n* (%)**			0.0001	**0.0004**
Lung	120 (64.5)	32 (66.7)		
Lymph nodes	55 (29.6)	19 (39.6)		
Bone	18 (9.7)	19 (39.6)		
Liver	13 (7.0)	5 (10.4)		
Other	24 (12.9)	0 (0.0)		
**ECOG performance status, *n* (%)**	*Missing: n = 1*	*Missing: n = 1*	<0.0001	**<0.0001**
0	42 (13.9)	1 (1.4)		
1	208 (68.9)	19 (26.4)		
2	41 (13.6)	26 (36.1)		
3	11 (3.6)	26 (36.1)		

[1] Chi-squared test; [2] Benjamini-Hochberg correction for multiple comparisons

Table S2. Chemotherapy regimens used as systemic treatment in R/M HNSCC patients during the one-year follow-up after diagnosis.

**First-line**	***n* = 303**	**Second-line (*Missing: n = 1*)**	***n* = 92**	**Third-line**	***n* = 16**	**Fourth-line**	***n* = 2**
**Chemotherapy, *n* (%)**	**269 (88.8)**	**Chemotherapy, *n* (%)**	**53 (58.2)**	**Chemotherapy, *n* (%)**	**10 (62.5)**	**Chemotherapy, *n* (%)**	**2 (100.0)**
Carboplatin	129	Paclitaxel	29	Paclitaxel	3	Paclitaxel	1
5-FU	124	Carboplatin	13	Methotrexate	3	Methotrexate	1
Paclitaxel	97	Methotrexate	10	Docetaxel	2		
Cisplatin	95	5-FU	7	Vinorelbine	1		
Methotrexate	19	Cisplatin	6	5-FU	1		
Docetaxel	18	Gemcitabine	3	Cisplatin	1		
Others	4	Docetaxel	2				
*With **Cetuximab***	*146 (54.3)*	*With **Cetuximab***	*16 (30.2)*	*With **cetuximab***	*0 (0.0)*	*With **cetuximab***	*0 (0.0)*
Cisplatin/5-FU	64	Paclitaxel	6				
5-FU/Carboplatin	39	Cisplatin/5-FU	4				
Carboplatin	11	Others	6				
Paclitaxel	10						
*With **Radiotherapy***	*51 (19.0)*	*With **Radiotherapy***	*4 (7.5)*	*With **Radiotherapy***	*0 (0.0)*	*With **Radiotherapy***	*0 (0.0)*
Cisplatin/5-FU + cetuximab	16	Paclitaxel	2				
Carboplatin/5-FU + cetuximab	8	Paclitaxel/Carboplatin	1				
Cisplatin	6	Cisplatin	1				
Others	21						
*With **Surgery***	*17 (6.3)*	*With **Surgery***	*1 (1.9)*	*With **Surgery***	*0 (0.0)*	*With **Surgery***	*0 (0.0)*
Cisplatin/5-FU + cetuximab	8	Carboplatin/5-FU + cetuximab	1				
Cisplatin	3						
Carboplatin/5-FU + cetuximab	1						
Paclitaxel/Carboplatin + cetuximab	1						
Others	4						
*With **Immunotherapy***	*2 (0.07)*	*With **Immunotherapy***	*0 (0.0)*	*With **Immunotherapy***	*0 (0.0)*	*With **Immunotherapy***	*0 (0.0)*
5-FU + cetuximab	1						
Cisplatin/5-FU + cetuximab	1						

Table S3. Duration, number of cycles/procedures, and dose of chemotherapy, cetuximab, anti-PD1 immunotherapy, radiotherapy, and surgery used in R/M HNSCC patients during the one-year follow-up after diagnosis.

Treatment modality	First-line	Second-line	Third-line
**Chemotherapy, mean ± SD**			
Carboplatin			
Duration (weeks)	14.3 ± 10.4	8.6 ± 6.3	-
Nr of cycles	4.6 ± 2.7	3.8 ± 1.8	-
Dose (mg)	471.3 ± 214.4	410.0 ± 217.0	-
5-FU			
Duration (weeks)	15.8 ± 9.9	9.1 ± 5.7	15.0 ± 0.0
Nr of cycles	5.0 ± 2.4	3.1 ± 2.0	4.0 ± 0.0
Dose (mg)	3016.3 ± 2375.1	3542.9 ± 1494.3	4000.0 ± 0.0
Paclitaxel			
Duration (weeks)	12.7 ± 9.6	10.8 ± 7.1	6.5 ± 6.4
Nr of cycles	6.3 ± 6.4	7.4 ± 5.5	4.0 ± 4.2
Dose (mg)	244.9 ± 126.4	149.9 ± 75.5	121.0 ± 35.6
Cisplatin			
Duration (weeks)	13.6 ± 7.6	6.0 ± 3.8	15.0 ± 0.0
Nr of cycles	4.6 ± 1.7	2.0 ± 0.9	4.0 ± 0.0
Dose (mg)	165.0 ± 160.6	139.0 ± 24.4	75.0 ± 0.0
Methotrexate			
Duration (weeks)	6.5 ± 4.4	6.3 ± 4.2	9.3 ± 4.6
Nr of cycles	6.5 ± 3.1	6.0 ± 4.0	9.0 ± 4.4
Dose (mg)	65.7 ± 5.9	53.3 ± 10.0	39.1 ± 7.9
Docetaxel			
Duration (weeks)	9.1 ± 6.4	8.0 ± 2.8	9.5 ± 7.8
Nr of cycles	3.5 ± 1.8	2.5 ± 0.7	3.5 ± 2.1
Dose (mg)	502.6 ± 1731.8	147.0 ± 0.0	220.0 ± 127.3
Gemcitabine			
Duration (weeks)	1.5 ± 0.7	1.3 ± 0.6	-
Nr of cycles	1.5 ± 0.7	1.0 ± 0.0	-
Dose (mg)	1456.0 ± 644.9	1200.0 ± 346.4	-
**Cetuximab, mean ± SD**			
Duration (weeks)	4.7 ± 13.8	7.6 ± 9.1	-
Nr of cycles	9.2 ± 10.0	6.6 ± 6.9	-
Dose (mg)	501.5 ± 184.3	421.1 ± 217.3	-
**Anti-PD1 immunotherapy, mean ± SD**			
Duration (weeks)	19.3 ± 16.2	13.1 ± 6.8	7.0 ± 3.3
Nr of cycles	8.4 ± 7.2	6.3 ± 3.4	3.8 ± 2.3
Dose (mg)	233.9 ± 70.0	263.4 ± 73.9	240.0 ± 0.0
**Radiotherapy, mean ± SD**			
Nr of sessions	18.4 ± 11.4	11.4 ± 11.3	13.0 ± 0.0
**Surgery, mean ± SD**			
Nr of surgeries	1.1 ± 0.3	1.0 ± 0.0	1.0 ± 0.0

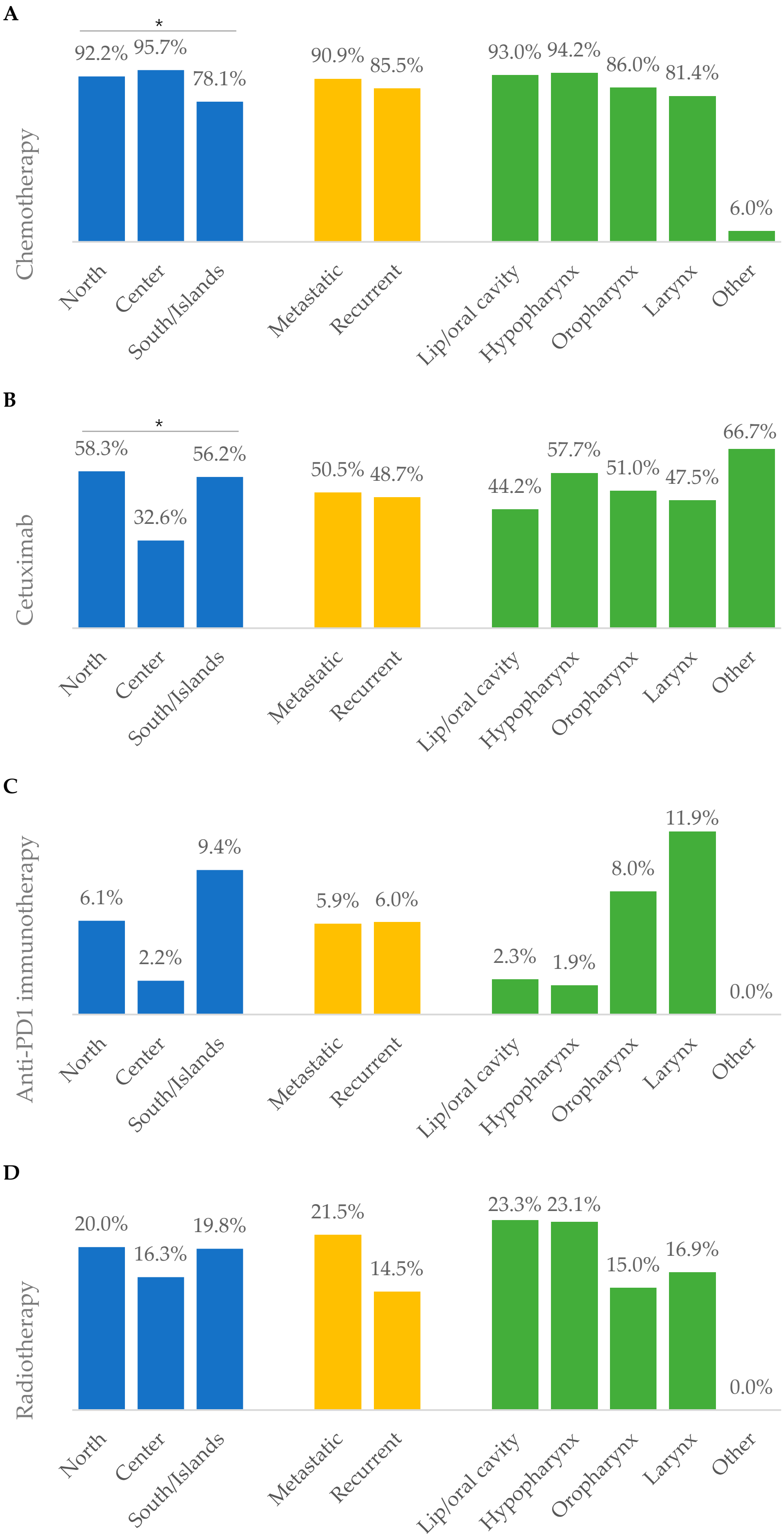
A
*
92.2%
95.7%
78.1%
90.9%
85.5%
93.0%
94.2%
86.0%
81.4%
6.0%
Chemotherapy
North
Center
South/Islands
Metastatic
Recurrent
Lip/oral cavity
Hypopharynx
Oropharynx
Larynx
Other
B
*
58.3%
32.6%
56.2%
50.5%
48.7%
44.2%
57.7%
51.0%
47.5%
66.7%
Cetuximab
North
Center
South/Islands
Metastatic
Recurrent
Lip/oral cavity
Hypopharynx
Oropharynx
Larynx
Other
C
6.1%
2.2%
9.4%
5.9%
6.0%
2.3%
1.9%
8.0%
11.9%
0.0%
Anti-PD1 immunotherapy
North
Center
South/Islands
Metastatic
Recurrent
Lip/oral cavity
Hypopharynx
Oropharynx
Larynx
Other
D
20.0%
16.3%
19.8%
21.5%
14.5%
23.3%
23.1%
15.0%
16.9%
0.0%
Radiotherapy
North
Center
South/Islands
Metastatic
Recurrent
Lip/oral cavity
Hypopharynx
Oropharynx
Larynx
Other

E

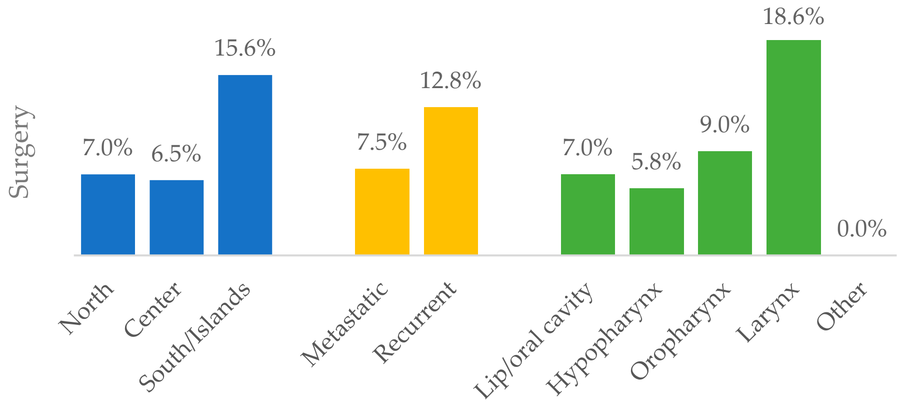

Figure S1. Use of first-line (A) chemotherapy, (B) cetuximab, (C) anti-PD1 immunotherapy, (D) radiotherapy, and (E) surgery by patient's geographical region (■), disease stage (■), and primary tumor location (■). *p = 0.002
